# Inconsistent effects of sleep deprivation on memory function

**DOI:** 10.17179/excli2021-3764

**Published:** 2021-06-04

**Authors:** Salar Vaseghi, Shirin Arjmandi-Rad, Gita Kholghi, Mohammad Nasehi

**Affiliations:** 1 Cognitive and Neuroscience Research Center (CNRC), Amir-Almomenin Hospital, Tehran Medical Sciences, Islamic Azad University, Tehran, Iran; 2 Department of Cognitive Neuroscience, Institute for Cognitive Science Studies (ICSS), Tehran, Iran; 3 Institute for Cognitive & Brain Sciences, Shahid Beheshti University, Tehran, Iran

**Keywords:** sleep deprivation, memory, learning, beneficial, destructive

## Abstract

In this review article, we aimed to discuss the role of sleep deprivation (SD) in learning and memory processing in basic and clinical studies. There are numerous studies investigating the effect of SD on memory, while most of these studies have shown the impairment effect of SD. However, some of these studies have reported conflicting results, indicating that SD does not impair memory performance or even improves it. So far, no study has discussed or compared the conflicting results of SD on learning and memory. Thus, this important issue in the neuroscience of sleep remains unknown. The main goal of this review article is to compare the similar mechanisms between the impairment and the improvement effects of SD on learning and memory, probably leading to a scientific solution that justifies these conflicting results. We focused on the inconsistent effects of SD on some mechanisms involved in learning and memory, and tried to discuss the inconsistent effects of SD on learning and memory.

## Introduction

In today's world, sleep deprivation (SD) is widespread. Unfortunately, the modern life restricts the essential time needed for sleep. There are many factors involved in SD including drinking alcohol, consuming caffeine, shift work, excessive light and noise, stress, anxiety, rumination, and some diseases (Medic et al., 2017[68]). Furthermore, sleep disorders including insomnia, narcolepsy, restless leg syndrome, and obstructive sleep apnea can lead to SD (Medic et al., 2017[68]). As we know, SD induces a wide-range of negative effects on cognitive functions (Rezaie et al., 2020[91][92]). Previous studies have shown that memory and mood state are highly affected following SD (Javad-Moosavi et al., 2020[45]; Kordestani-Moghadam et al., 2020[52]; Mahdavi et al., 2020[65]). But why do sleep restrictions dramatically disrupt many of the cognitive and physiological processes? To answer this question, we can point to a neurobiological perspective of SD (Krause et al., 2017[55]): It is very important to characterize which brain networks are vulnerable or resilient to the effects of SD and to understand how SD-induced alterations (changes in the activity of different brain areas or their connectivity) justify the maladaptive changes in cognitive functions related to SD. SD induces selective effects on certain brain structures and functions, while there are individual differences in the effects of SD (Alhola and Polo-Kantola, 2007[4]). As mentioned, SD strongly disrupts memory processing. But interestingly, many studies have shown conflicting results. Some studies have reported the beneficial effects of SD on learning and memory, and some studies have not shown any destructive effect. This is an important dilemma, and the main goal of the present review is to discuss these contradictory effects. We need to reach a clear position on SD and its effects on learning and memory processing. Due to the increasing prevalence of SD in our societies, there is a need to discover what really are the effects of SD on memory function? Thus, at first, we discuss the effects of SD on different types of memory in basic and clinical studies.

## SD and Memory

### Impairment effects of SD on learning and memory

There are many studies reporting the impairment effect of total SD (TSD) or rapid-eye movement SD (RSD) on learning and memory. In this review, we do not mention all these studies; This is a repetitive work. Thus, we declare some of these studies. Numerous studies have shown the adverse effects of SD on learning and memory (Chen et al., 2020[18]; Zhang et al., 2020[125]). It has been shown that TSD or RSD for 24 hours disrupts passive avoidance memory acquisition in rats (Eydipour et al., 2020[23]; Javad-Moosavi et al., 2020[45]). 24 hours TSD impairs memory performance in the shuttle box apparatus in rats (Rezaie et al., 2020[91]). 24 hours TSD also impairs social interaction memory in rats (Almaspour et al., 2020[5], Rezaie et al., 2020[92]). Furthermore, 48 hours TSD impairs fear-conditioning memory in rats (Kordestani-Moghadam et al., 2020[51]). 24 hours TSD or RSD also attenuates memory formation in the inhibitory passive avoidance apparatus in rats (Javad-Moosavi et al., 2017[46]). 12, or 24-, or 36-hours RSD impairs contextual memory retrieval, while 24- or 36-hours RSD impairs auditory fear memory retention (Nasehi et al., 2019[77]). Previous research has declared that sleep is essential not only for acquisition and consolidation but also for the retrieval of fear memories, indicating 24 hours TSD impairs fear memory retrieval (Montes-Rodriguez et al., 2019[71]). It has been reported that 14-day chronic SD impairs spatial memory, object location recognition memory, and novel object recognition memory in rats (Tang et al., 2020[105]). Previous research has also shown that TSD decreases working memory capacity (Peng et al., 2020[84]). RSD impairs spatial memory performance in rats (Ocalan et al., 2019[79]). 72 hours RSD induces an impairment effect on spatial learning and memory of rats in the Morris water maze apparatus (Wang et al., 2009[117]). Chronic RSD also impairs short- and long-term memory in the radial arm water maze task (Alzoubi et al., 2017[9]). Also, chronic TSD impairs short- and long-term memory in the radial arm water maze task (Alzoubi et al., 2018[8]). Brief periods of SD can disrupt consolidation of hippocampus-dependent associative and spatial learning in rodents (Smith and Rose, 1996[102]; Graves et al., 2003[32]). It has been declared that SD suppresses neurogenesis induced by hippocampus-dependent learning in rats (Hairston et al., 2005[36]). The negative interaction effects of chronic and acute SD on spatial working memory in human have been shown in a recent study (Hennecke et al., 2020[41]). It has been shown that acute SD induces weakly encoded memories in human subjects (Baena et al., 2020[11]). 24 hours SD also impairs working memory in healthy adults (Guo et al., 2019[33]). TSD for 24 hours also impairs working memory in healthy young men (Sauvet et al., 2020[95]) (Table 1(Tab. 1); References in Table 1: Almaspour et al., 2020[5]; Alzoubi et al., 2017[9], 2018[8]; Eydipour et al., 2020[23]; Guo et al., 2019[33]; Hennecke et al., 2020[41]; Javad-Moosavi et al., 2017[46], 2020[45]; Kordestani-Moghadam et al., 2020[51]; Montes-Rodriguez et al., 2019[71]; Nasehi et al., 2019[77]; Ocalan et al., 2019[79]; Rezaie et al., 2020[91][92]; Sauvet et al., 2020[95]; Tang et al., 2020[105]; Wang et al., 2009[117]).

### Improvement (or no impairment) effects of SD on learning and memory

On the contrary, there are some reports showing the improvement effect of SD on learning and memory. We prefer to focus on these results, according to the goal of the present review. For example, it has been reported that 24 hours acute SD improves learning and memory in splenectomized rats (Zhang et al., 2020[127]). Short-term SD ameliorates the impairments in learning and memory in rats following global cerebral ischemia/reperfusion (Cheng et al., 2015[19]). 24 hours RSD also improves spatial memory impairment in male Wistar rats (Mahboubi et al., 2019[64]). Interestingly, a previous study has reported that short-term SD stimulates hippocampal neurogenesis in rats (Cheng et al., 2015[19]). In addition, in a previous study, 12 hours short-term SD promoted neurogenesis in the hippocampus of rats (Junek et al., 2010[48]). Another study has shown that one night SD stimulates neurogenesis in the dentate gyrus of rats via increasing cell proliferation and survival of newly generated cells, while SD has no effect on the number of newly generated cells in the subventricular zone of the lateral ventricles, suggesting a region-specific response to SD (Grassi Zucconi et al., 2006[31]). Importantly, it has been suggested that SD, less than 24 hours, may actually increase cell proliferation (Mueller et al., 2015[76]). It has been also revealed that acute RSD improves avoidance learning and spatial memory in rats (Azogu et al., 2015[10]). Post-learning paradoxical SD also enhances avoidance performance in rats (Gisquet-Verrier and Smith, 1989[30]). In addition, a previous study has shown that acquisition of memory is not affected by SD in honeybees (Hussaini et al., 2009[42]). SD during several days has no effect on long-term recall of declarative and procedural memories in adolescents (Voderholzer et al., 2011[114]). Another related study has shown that acute SD does not affect declarative memory in 10‐year‐old girls (Biggs et al., 2010[12]). Furthermore, verbal working memory and declarative memory are not impaired following SD (Hennecke et al., 2020[41]). SD does not impair attention, response inhibition capacity, and working memory performance in human subjects (Giacobbo et al., 2016[29]). SD also impairs objective but not self-estimated working memory performance in women, whereas both are not changed following SD in men (Rangtell et al., 2019[89]). It has also been shown that slow wave sleep restriction or RSD does not affect sleep-dependent memory consolidation (Genzel et al., 2009[27]). RSD does not impair spatial learning in the Morris water maze task in rats (Cakir et al., 2020[15]). A previous study has reported that RSD does not block re-consolidation of fear conditioning (Tian et al., 2009[107]). Also, RSD does not impair emotional verbal memory in young healthy subjects (Morgenthaler et al., 2014[73]). Some studies have reported that RSD does not impair fear memory (Silvestri, 2005[100], Silvestri and Root, 2008[101]). Importantly, SD increases the activation of the amygdala in response to emotional stimuli, due to a disconnection between the amygdala and the prefrontal cortex, leading to a potential enhancement effect on negative emotional memory (Yoo et al., 2007[123]). Furthermore, SD induces a similar hyper-responsivity of the amygdala to emotional faces (increase of negative emotional reaction) as well as reduce its functional connectivity with the ventral anterior cingulate cortex (Motomura et al., 2013[74]). It has also been shown that sleep restriction enhances the activation of the amygdala for subliminal signals of fear, indicating that SD enhances subliminal emotion processing that engages the amygdala and its connection to the superior colliculus (Motomura et al., 2014[75]). Another research has shown an increase in behavioral and neural reactivity to emotional images in RSD subjects (Rosales-Lagarde et al., 2012[93]). Furthermore, compared to young controls, sleep-deprived young adults show intact recognition rates for negative emotion memories (Vargas et al., 2019[110]). Interestingly, the left frontal, the right frontal, and the left parietal lobes are more active after TSD or early night SD, leading to higher scores for word retrieval in students (Tantawy et al., 2013[106]). A previous study has reported that SD shows conflicting results for working memory performance, indicating some studies have shown a decrease in working memory performance, and other studies have reported no effect (de Bruin et al., 2017[20]). A better performance in a verbal memory test in sleep restricted children has also been shown in a past study (Randazzo et al., 1998[88]) (Table 2(Tab. 2); References in Table 2: Azogu et al., 2015[10]; Biggs et al., 2010[12]; Cakir et al., 2020[15]; Cheng et al., 2015[19]; Giacobbo et al., 2016[29]; Gisquet-Verrier and Smith, 1989[30]; Hennecke et al., 2020[41]; Hussaini et al., 2009[42]; Mahboudi et al., 2019[64]; Morgenthaler et al., 2014[73]; Randazzo et al., 1998[88]; Rangtell et al., 2019[89]; Voderholzer et al., 2011[114]; Zhang et al., 2020[127]).

## The Mechanisms Underlying the Effects of SD on Learning and Memory

### Mechanisms underlying destructive effects

As mentioned, SD usually impairs learning and memory processing. SD induces a deleterious effect on proliferation of newly born neuronal cells in the hippocampus (Meerlo et al., 2009[69]), and this effect can lead to attenuated memory formation. It has been shown that SD significantly reduces neurogenesis in the hippocampus, leading to memory decline (Wadhwa et al., 2019[115]). A previous research has reported that SD induces shrinkage and loss of hippocampal neurons in mice, and impairs novel object recognition and object location memories (Wang et al., 2020[120]). Furthermore, SD disturbs hippocampal neuronal excitability in young APP/PS1 mice and impairs spatial memory performance (Tabassum et al., 2019[103]). The memory impairment effect of SD through attenuating hippocampal neurogenesis has also been reported (Guzman-Marin et al., 2005[34]; Lopez-Virgen et al., 2015[62]). 48 hours RSD reduces viable neurons in the hippocampus of mice and impairs memory performance in the Y-maze task (Olonode et al., 2019[80]). Furthermore, SD reduces neuronal cell proliferation and differentiation (Wadhwa et al., 2017[116]). SD highly affects a wide-range of hippocampal signaling pathways including transcriptional and translational processes (Vecsey et al., 2012[112]; Tudor et al., 2016[109]). Interestingly, SD dramatically affects hippocampal memory consolidation in the first few hours following training, when it overlaps with the second wave of cAMP (cyclic adenosine monophosphate) signaling, transcription, and protein synthesis critical for enhancing synaptic plasticity and memory storage (Havekes and Abel, 2017[38]). For example, 3 hours SD commencing 1 hour after training impairs the formation of spatial memory, while 3 hours after training do not alter memory consolidation (Prince et al., 2014[85]). SD decreases cAMP levels in the CA1 region of the hippocampus (Vecsey et al., 2009[111]). SD can also induce changes in second messenger pathways such as the cAMP-PKA (protein kinase A) signaling pathway (Florian et al., 2011[24]) and synaptic structure (Havekes et al., 2016[40]). It should be noted that, restoring cAMP levels in the hippocampus reverses sleep deprivation-induced memory impairments (Havekes and Abel, 2017[38]).

In addition, SD attenuates LTP (long-term potentiation) (Rezaie et al., 2020[91]). LTP is a critical mechanism in memory processes that modulates synaptic transmission especially in the hippocampus (McDermott et al., 2003[67]). SD disturbs cAMP-dependent forms of synaptic plasticity such as long-lasting forms of LTP and memory consolidation (Vecsey et al., 2009[111]; Havekes et al., 2014[39]). It has been reported that 72 hours TSD reduces LTP, attenuates synaptic plasticity, and impairs learning and memory in female rats (Rajizadeh et al., 2020[87]). RSD significantly reduces LTP induction in the hippocampus (Ishikawa et al., 2006[44]). TSD or RSD highly attenuates synaptic plasticity through decrease in PKA activity and phosphorylation of CREB (critical for long-term synaptic plasticity) in the hippocampus (Alhaider et al., 2011[3]). It has been reported that SD reduces neuronal plasticity in the hippocampus by decreasing intracellular cAMP-PKA signaling, leads to changes in CREB-mediated gene transcription, neurotrophic signaling, and glutamate receptor expression (Kreutzmann et al., 2015[56]). CREB is essential for hippocampal synaptic plasticity and LTP, and SD alters its level in the hippocampus, the striatum, and the cortex (Wang et al., 2010[118]; Abel et al., 2013[1]; Duncan et al., 2013[22]). It has been shown that CREB phosphorylation is decreased after 5 to 6 hours of TSD or RSD in the hippocampus (Vecsey et al., 2009[111]; Zhao et al., 2010[128]). SD selectively impairs PKA-dependent forms of LTP in the hippocampus (Vecsey et al., 2009[111]). A previous study has demonstrated that maternal SD attenuates LTP in the CA1 hippocampal region and reduces basal synaptic transmission (Peng et al., 2016[83]). The impairment effect of SD on LTP may be related to the disruption of cAMP signaling (Vecsey et al., 2009[111]). 

SD also aggravates oxidative stress and inflammation. SD increases oxidized glutathione levels, reduced oxidized glutathione/glutathione ratio, and diminishes catalase and glutathione peroxidase activity, leading to hippocampal oxidative stress (Silva et al., 2004[99]; Alzoubi et al., 2012[7]). It has been reported that 72 hours SD significantly increases the production of ROS (reactive oxygen species) in the hippocampus (Salehpour et al., 2018[94]). Also, SD increases oxidative stress in the hippocampus via attenuating the activation of SOD (superoxide dismutase) (Zhang et al., 2013[126]). Acute SD induces oxidative stress and subsequent memory impairments (Aleisa et al., 2011[2]). A previous research has shown that SD impairs object location recognition and passive avoidance memories via increasing oxidative stress in the brain (Zhou et al., 2020[129]). It has also been revealed that 5-day TSD induces spatial memory decline in the Morris water maze task through increasing oxidative stress in the hippocampus of rats (Wang et al., 2018[119]). RSD via increasing ROS during wakefulness and induction of oxidative stress impairs memory function (Alzoubi et al., 2017[9], 2019[6]). Furthermore, SD increases pro-inflammatory cytokines in the hippocampus (Wadhwa et al., 2017[116]). SD can also impair memory performance via induction of autophagy in the hippocampus (Yang et al., 2019[122]). Other studies have shown that SD induces autophagy and apoptosis in the hippocampus (Cao et al., 2019[16], 2020[17]). 

Studies have shown that SD reduces the level of BDNF (brain-derived neurotrophic factor) in the brain (Rahmani et al., 2020[86]). BDNF increases the frequency of miniature excitatory postsynaptic currents (EPSCs) in the brain (Binder and Scharfman, 2004[13]). The critical role of BDNF in the induction of LTP has also been revealed (Korte et al., 1996[54]; Patterson et al., 1996[81]). It has been shown that BDNF knockout animals show LTP impairment (Korte et al., 1995[53]). BDNF significantly modulates both sleep and memory (Mascetti et al., 2013[66]; Giacobbo et al., 2016[29]). A previous study has shown that 24 hours RSD reduces BDNF levels in the hippocampus and the prefrontal cortex (Mahboubi et al., 2019[64]). TSD downregulates BDNF expression in the hippocampus and impairs spatial memory formation in rats (Duan et al., 2016[21]). The expression of BDNF and its receptor, TrkB (tropomyosin receptor kinase) is decreased following short-term SD in the hippocampus and the amygdala of mice, leading to attenuated memory retrieval (Sharma et al., 2020[96]). Another study has shown that 7-day SD reduces the level of BDNF and TrkB in the hippocampus of mice, leading to impaired memory performance in the step-down avoidance and the Morris water maze tests (Hwang et al., 2019[43]). 24 hours SD decreases the level of BDNF in the CA1 region of the hippocampus in rats and impairs long-term memory (Zagaar et al., 2013[124]). Also, in sleep-deprived university students, BDNF plasma level is significantly lower than in normal students (Kuhn et al., 2016[58]).

### Mechanisms underlying beneficial effects

The reasons beyond the beneficial effects of SD on learning and memory are ambiguous. Although most studies have shown that SD impairs learning and memory, some conflicting results on the effect of SD on learning and memory have been reported. One of the inconsistent findings in related studies is the different impact of SD on cognitive capability and expression of some neurochemicals in various regions of the brain (Mahboubi et al., 2019[64]). Interestingly, it has been reported that perioperative sleep fragmentation highly elevates hippocampal inflammation without further memory impairment, while sleep fragmentation or surgery independently induces a significant memory impairment effect (Zhang et al., 2020[125]). It has been shown that SD via increasing IL-6 (interleukin-6) improves learning and memory performance, indicating elevated IL-6 decreases tau phosphorylation through increasing FOXQ1 in the hippocampus (Zhang et al., 2020[127]). FOXQ1 is the direct target gene of miR-125b, involved in neural apoptosis and the phosphorylation of tau (Ma et al., 2017[63]). In fact, SD can indirectly improve memory performance. Importantly, the relation between IL-6 and SD is so controversial. For example, it has been shown that IL-6 level is similar between control and SD subjects (Vgontzas et al., 2002[113]). Moreover, a significant shift of the major peak of IL-6 secretion from midnight (4 a.m.) to evening (7 p.m.) has been revealed (Vgontzas et al., 2002[113]). However, a previous study has shown that sleep onset is related to an increase in serum levels of IL-6 (Redwine et al., 2000[90]). It has also been shown that different stages of sleep have a different effect on IL-6 serum level (Redwine et al., 2000[90]). In healthy sleep-deprived volunteers, the level of IL-6 in the plasma is increased (Haack et al., 2007[35]). However, it has been shown that 6-12 hours short-term SD prior to cerebral ischemia induces neuroprotective effects via decreasing inflammatory responses and glial reactions in the hippocampus of rats (Weil et al., 2009[121]; Moldovan et al., 2010[70]). It seems that the effect of SD on inflammation may significantly depend on the time window and duration of SD (Cheng et al., 2015[19]).

A previous study has shown that short-term SD increases the level of BDNF in the hippocampus of rats (Cheng et al., 2015[19]). Other studies have also shown that 12 hours short-term SD increases the expression level of hippocampal BDNF (Fujihara et al., 2003[25]; Hairston et al., 2004[37]). Furthermore, 24 hours RSD enhances the level of BDNF and TrkB in the hippocampus and the prefrontal cortex of rats exposed to intensive exercise, leading to memory improvement (Mahboubi et al., 2019[64]). In line with these results, it has been shown that RSD increases the level of BDNF in the hippocampus (Jiang and Zhu, 2015[47]). In a previous study, the sleep deprived human subjects showed higher BDNF levels and normal performance on attention, response inhibition capacity, and working memory (Giacobbo et al., 2016[29]). It has also been shown that 8 hours TSD does not change the level of BDNF in rats (Taishi et al., 2001[104]). Therefore, the inconsistent effects of SD on the level of BDNF can be involved in various effects of SD on memory performance. Sleep homeostatic and circadian mechanisms referring to the two process models of sleep regulation can be a suggested mechanism that is associated with the enhancement effect of SD on the BDNF level in the hippocampus (Kavcic et al., 2011[49]; Mahboubi et al., 2019[64]). 

Note that, at least 10 genes needed for mammalian circadian clock function have been identified (Kavcic et al., 2011[49]). The positive modulators in mammals are *Clock* and *Bmal1*, while *Per1-3, Cry1-2, *and* Dec1-2* are involved in the negative feedback loop (Shearman et al., 2000[97]; Gachon et al., 2004[26]). 40 hours acute SD affects the expression of *hPer2* (a clock gene) in PBMCs (peripheral blood mononuclear cells) and leads to circadian rhythm disturbances (Kavcic et al., 2011[49]). A significant variation in *hPer2* and *hBmal1* expression levels under SD conditions has been reported in a previous study (Kavcic et al., 2011[49]). Importantly, BDNF-mediated signaling has an important role in the regulation of circadian rhythm (Liang et al., 2000[59]). BDNF modulates circadian pacemaker function via its localization in the SCN (Liang et al., 1998[60]). The relation between the level of BDNF and circadian gene expression has also been revealed (Moravcova et al., 2020[72]). It has been reported that BDNF is rhythmically expressed within the SCN, indicating its level is increased during the subjective night, when light shifts the phase of circadian rhythms, but is low throughout the subjective day (Liang et al., 1998[61]). BDNF plays a critical role in the time-dependent regulation of the circadian rhythm by light (Liang et al., 2000[59]). BDNF level is significantly related to obesity and neurodegeneration, and these pathologies are characterized by disrupted circadian rhythm (Genzer et al., 2016[28]). A previous finding has shown that disrupted circadian rhythm reduces the expression of BDNF and TrkB in the hippocampus of mice (Kim et al., 2019[50]). Whereas, another study has shown that *clock* knockdown in hippocampal neurons upregulates BDNF mRNA by 86 % (Genzer et al., 2016[28]). Importantly, it should be noted that IL-6 exhibits a circadian rhythm (Nilsonne et al., 2016[78]), and is known as a possible sleep regulatory substance (Krueger 2008[57]). As mentioned, the effect of SD on IL-6 can be a possible mechanism for the improvement effect of SD on memory function.

It has also been suggested that heightened arousal and/or SD-induced stress can have a role in the improvement effect of SD on memory (Azogu et al., 2015[10]). Acute stress can enhance associative learning in rodents (Shors, 2001[98]). Interestingly, SD can induce a stress and heighten arousal via activating the *locus coeruleus* noradrenergic system, leading to sensitized postsynaptic noradrenergic receptors in critical neuroplastic circuits, which in turn, facilitates arousal and augments attentional processes (Payne et al., 2002[82]). Furthermore, it has been shown that repeated periods of 4 hours SD during five days have no effect on avoidance learning, suggesting that animals can adopt to this condition via sleep rebound over the rest of the day (Borbely et al., 1984[14]; Tobler and Borbely, 1990[108]). Additionally, as previously mentioned, a previous study has reported that short-term SD stimulates hippocampal neurogenesis in rats (Cheng et al., 2015[19]). Another study has also shown this effect (Grassi Zucconi et al., 2006[31]); although more studies are needed, but this effect can lead to a better memory performance and a better mood state (Grassi et al., 2006[31]) (Table 3(Tab. 3); References in Table 3: Cheng et al., 2015[19]; Duan et al., 2016[21]; Fujihara et al., 2003[25]; Giacobbo et al., 2016[29]; Grassi Zucconi et al., 2006[31]; Guzman-Marin et al., 2005[34]; Hairston et al., 2004[37], 2005[36]; Hwang et al., 2019[43]; Junek et al., 2010[48]; Kavcic et al., 2011[49]; Kuhn et al., 2016[58]; Mahboubi et al., 2019;[64] Olonode et al., 2019[80]; Sharma et al., 2020[96]; Taishi et al., 2001[104]; Wadhwa et al., 2019[115]; Wang et al., 2020[120]; Zagaar et al., 2013[124]).

## Concluding Remarks and Future Perspective

Eventually, an important question remains about the role of SD in memory processing: Is SD destructive or beneficial for memory function? As mentioned throughout the manuscript, most of the studies have shown the impairment effects of SD (TSD and RSD) on learning and memory performance. However, some studies have shown the opposite results, indicating that SD improves learning and memory. This is a critical dilemma. We should find the biological and cellular reasons beyond these inconsistent results. We mentioned to several mechanisms underlying the impairment effect of SD on memory processing. We also mentioned to some mechanisms underlying the improvement effect of SD on memory processing. Thus, we should focus on similarities between these conflicting studies. These similarities are as follows: 1) Neurogenesis: as mentioned, SD may induce dualistic effects on neurogenesis in the hippocampus. SD has shown both improvement and impairment effects on neurogenesis in the hippocampus. On the other hand, neurogenesis induces a region-specific response to SD. Importantly, as Table 3(Tab. 3) shows, it seems that short-term SD stimulates neurogenesis, while long-term SD induces an impairment effect, concluding that the duration of SD plays an important role in cell proliferation changes following SD. Thus, it seems that the duration of SD may have a critical role in the modulation of neurogenesis. However, more detailed studies are needed to prove this point. 2) BDNF: as mentioned, BDNF has a critical role in the effects of SD on memory. On the other hand, SD can alter the expression level of BDNF and its receptor, TrkB. Many studies have shown that SD reduces the expression of BDNF, while some studies have shown the opposite result. The upregulation of BDNF can trigger the neurogenesis in the hippocampus, leading to a better memory performance. Importantly, disrupted circadian rhythm following SD can affect the expression level of BDNF. Thus, the effect of SD or disrupted circadian rhythm on the expression level of BDNF and subsequent neurogenesis may have an important role in the impact of SD on memory processing. 3) Circadian genes: as mentioned, BDNF-mediated signaling plays a crucial role in circadian regulation. Also, SD significantly affects the normal rhythm of the circadian cycle. Disrupted circadian rhythm and any disturbances in the expression of circadian genes can induce both increment or decrement effect on the BDNF level, leading to inconsistent effects on memory. 4) IL-6: as mentioned, the relation between IL-6 and SD is so controversial. Inflammation plays a critical role in response to SD. During SD, the level of IL-6 shows a variation and its change depends on the time. SD also induces inconsistent effects on the level of IL-6, leading to various effects on memory function. Importantly, the effect of SD on inflammation significantly depends on the time window and duration of SD. Also, the role of IL-6 in modulating circadian rhythm is so important and should be considered in future studies. 5) Stress: as mentioned, heightened arousal and/or SD-induced stress can have a role in the improvement effect of SD on memory. However, this stress can also induce an impairment effect on memory performance, because stress by itself, can induce an adverse effect on neurogenesis. Also, adaptation of animals to SD condition may alter their memory performance and neutralize the adverse effect of stress on memory function. Thus, the role of SD-induced stress is so important and can dramatically affect learning and memory performance.

## Conflict of interest

The authors declare that they have no conflict of interest.

## Funding information

There is no providing financial support to this project.

## Figures and Tables

**Table 1:**
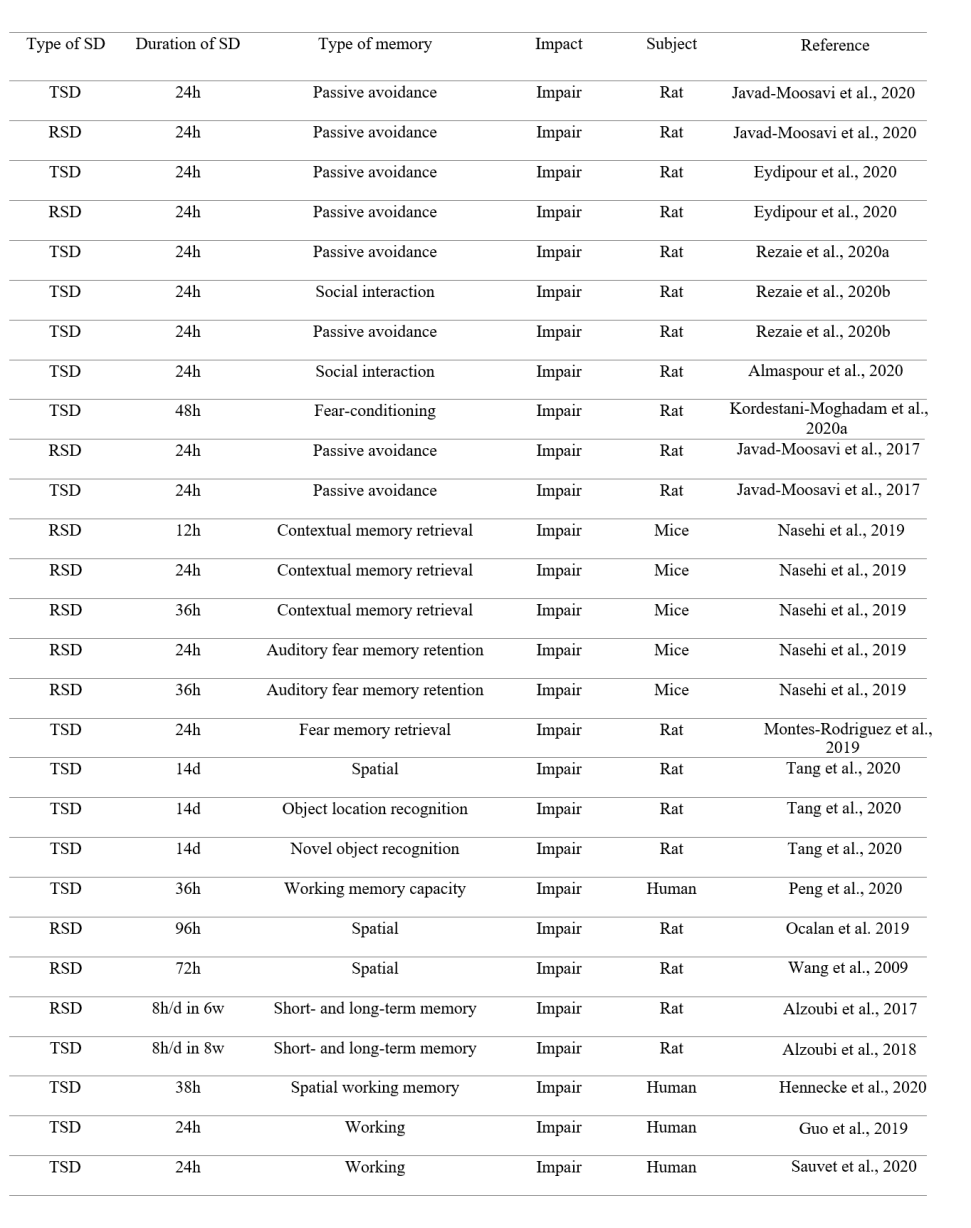
The impairment effect of TSD or RSD on different types of memory (h = hour, d = day, w = week, SD = sleep deprivation, TSD = total sleep deprivation, RSD = REM sleep deprivation)

**Table 2:**
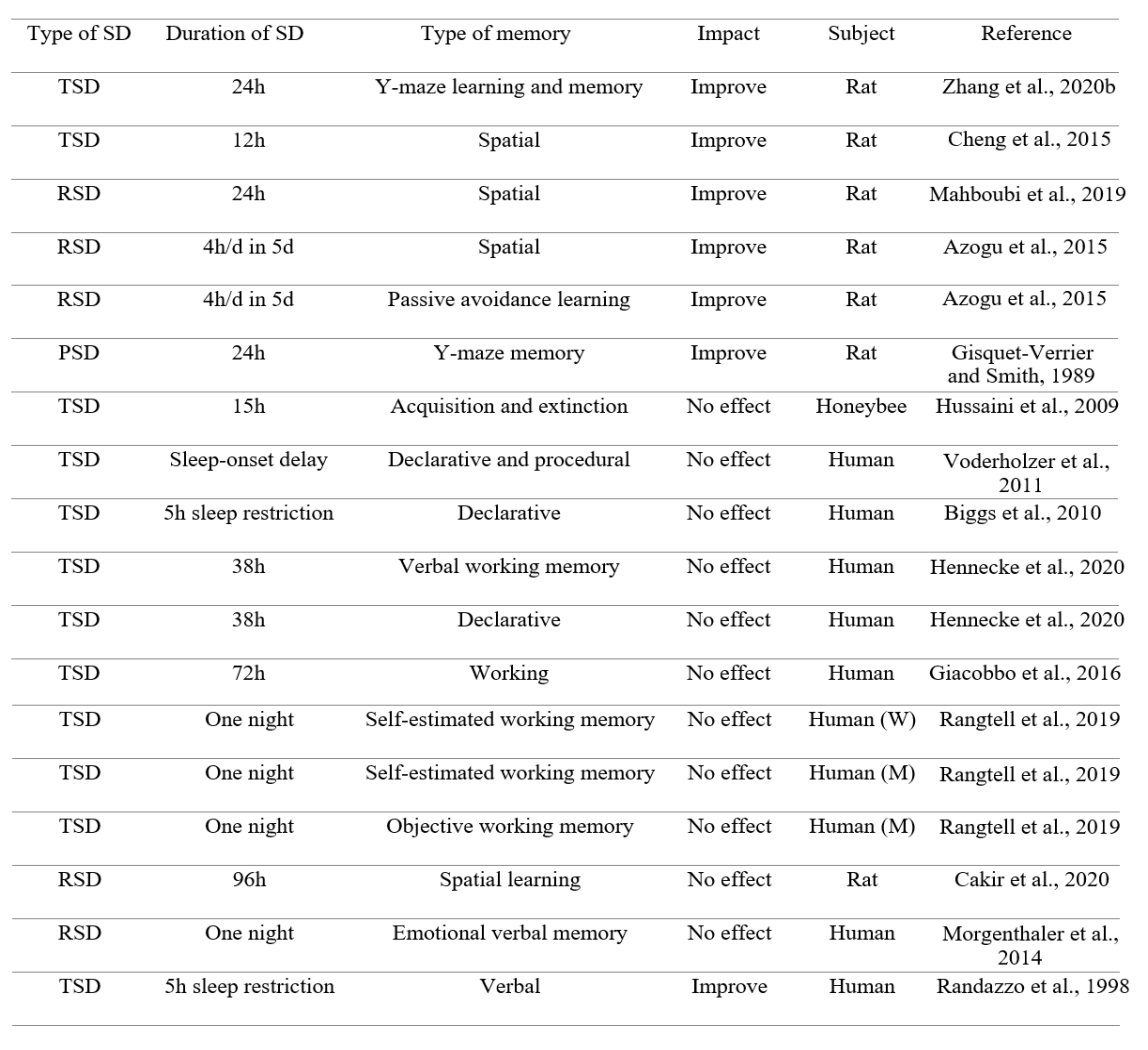
The improvement (or no impairment) effect of TSD or RSD on different types of memory (h = hour, d = day, SD = sleep deprivation, TSD = total sleep deprivation, RSD = REM sleep deprivation, PSD = paradoxical sleep deprivation, M = Men, W = Women)

**Table 3:**
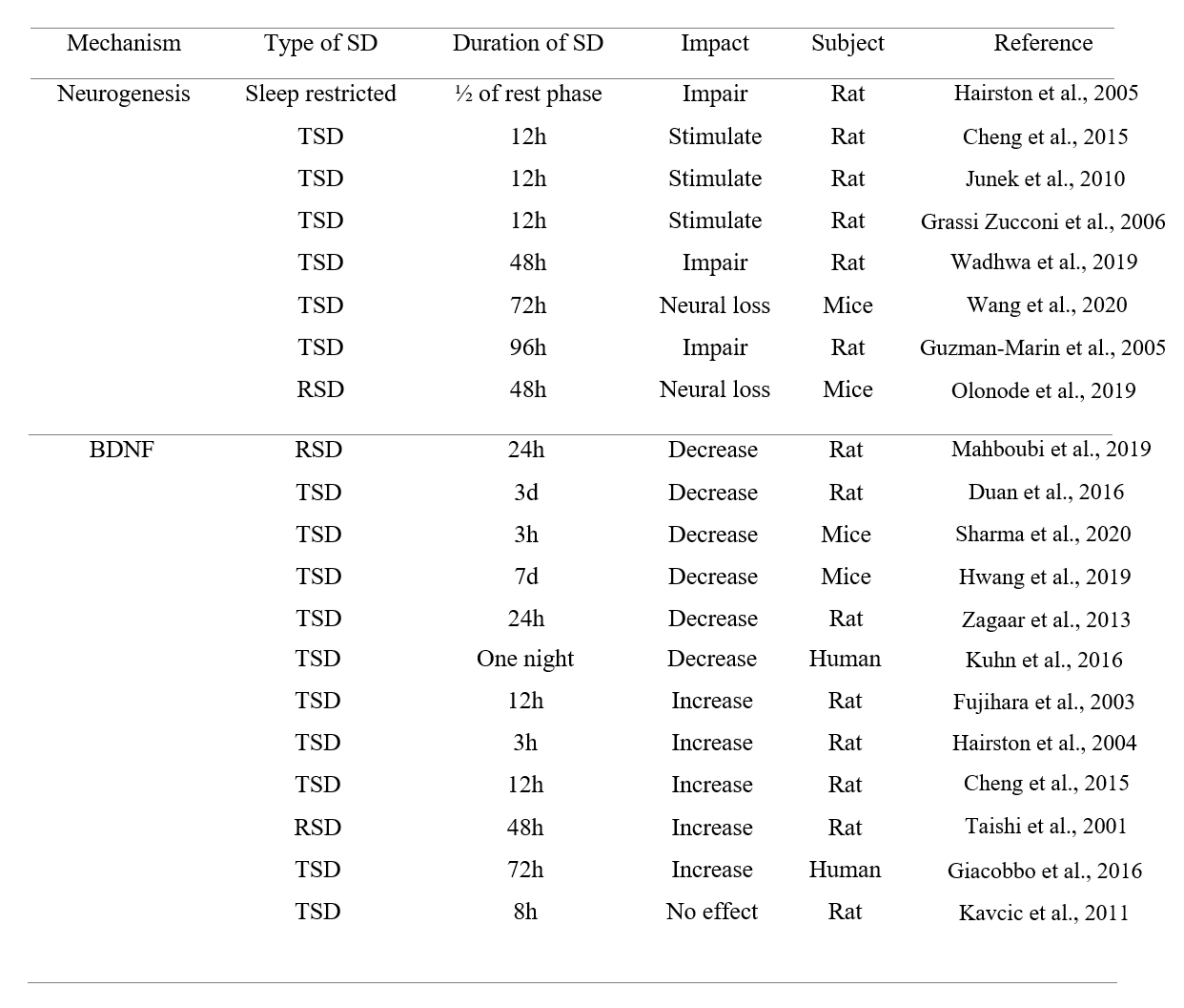
The inconsistent effects of TSD or RSD on neurogenesis and BDNF level (h = hour, d = day, SD = sleep deprivation, TSD = total sleep deprivation, RSD = REM sleep deprivation)

## References

[1] Abel T, Havekes R, Saletin JM, Walker MP (2013). Sleep, plasticity and memory from molecules to whole-brain networks. Curr Biol.

[2] Aleisa AM, Helal G, Alhaider IA, Alzoubi KH, Srivareerat M, Tran TT (2011). Acute nicotine treatment prevents REM sleep deprivation-induced learning and memory impairment in rat. Hippocampus.

[3] Alhaider IA, Aleisa AM, Tran TT, Alkadhi KA (2011). Sleep deprivation prevents stimulation-induced increases of levels of P-CREB and BDNF: protection by caffeine. Mol Cell Neurosci.

[4] Alhola P, Polo-Kantola P (2007). Sleep deprivation: Impact on cognitive performance. Neuropsychiatr Dis Treat.

[5] Almaspour MB, Nasehi M, Khalifeh S, Zarrindast MR (2020). The effect of fish oil on social interaction memory in total sleep-deprived rats with respect to the hippocampal level of stathmin, TFEB, synaptophysin and LAMP-1 proteins. Prostaglandins Leukot Essent Fatty Acids.

[6] Alzoubi KH, Al Subeh ZY, Khabour OF (2019). Molecular targets for the interactive effect of etazolate during post-traumatic stress disorder: Role of oxidative stress, BDNF and histones. Behav Brain Res.

[7] Alzoubi KH, Khabour OF, Rashid BA, Damaj IM, Salah HA (2012). The neuroprotective effect of vitamin E on chronic sleep deprivation-induced memory impairment: the role of oxidative stress. Behav Brain Res.

[8] Alzoubi KH, Malkawi BS, Khabour OF, El-Elimat T, Alali FQ (2018). Arbutus andrachne L. Reverses sleep deprivation-induced memory impairments in rats. Mol Neurobiol.

[9] Alzoubi KH, Rababa'h AM, Owaisi A, Khabour OF (2017). L-carnitine prevents memory impairment induced by chronic REM-sleep deprivation. Brain Res Bull.

[10] Azogu I, de la Tremblaye PB, Dunbar M, Lebreton M, LeMarec N, Plamondon H (2015). Acute sleep deprivation enhances avoidance learning and spatial memory and induces delayed alterations in neuro-chemical expression of GR, TH, DRD1, pCREB and Ki67 in rats. Behav Brain Res.

[11] Baena D, Cantero JL, Fuentemilla L, Atienza M (2020). Weakly encoded memories due to acute sleep re-striction can be rescued after one night of recovery sleep. Sci Rep.

[12] Biggs SN, Bauer KMM, Peters J, Dorrian J, Kennedy JD, Martin AJ (2010). Acute sleep restriction does not affect declarative memory in 10-year-old girls. Sleep Bioll Rhythms.

[13] Binder DK, Scharfman HE (2004). Brain-derived neurotrophic factor. Growth Factors.

[14] Borbely AA, Tobler I, Hanagasioglu M (1984). Effect of sleep deprivation on sleep and EEG power spectra in the rat. Behav Brain Res.

[15] Cakir A, Ocalan B, Koc C, Suyen GG, Cansev M, Kahveci N (2020). Effects of CDP-choline administration on learning and memory in REM sleep-deprived rats. Physiol Behav.

[16] Cao Y, Li Q, Liu L, Wu H, Huang F, Wang C (2019). Modafinil protects hippocampal neurons by suppressing excessive autophagy and apoptosis in mice with sleep deprivation. Br J Pharmacol.

[17] Cao Y, Yang Y, Wu H, Lu Y, Wu S, Liu L (2020). Stem-leaf saponins from Panax notoginseng coun-teract aberrant autophagy and apoptosis in hippocampal neurons of mice with cognitive impairment induced by sleep deprivation. J Ginseng Res.

[18] Chen D, Zhang Y, Wang C, Wang X, Shi J, Zhang J (2020). Modulation of hippocampal dopamine and synapse-related proteins by electroacupuncture improves memory deficit caused by sleep deprivation. Acupunct Med.

[19] Cheng O, Li R, Zhao L, Yu L, Yang B, Wang J (2015). Short-term sleep deprivation stimulates hippocampal neurogenesis in rats following global cerebral ischemia/reperfusion. PLoS One.

[20] de Bruin EJ, van Run C, Staaks J, Meijer AM (2017). Effects of sleep manipulation on cognitive functioning of adolescents: A systematic review. Sleep Med Rev.

[21] Duan R, Liu X, Wang T, Wu L, Gao X, Zhang Z (2016). Histone acetylation regulation in sleep deprivation-induced spatial memory impairment. Neurochem Res.

[22] Duncan WC, Sarasso S, Ferrarelli F, Selter J, Riedner BA, Hejazi NS (2013). Concomitant BDNF and sleep slow wave changes indicate ketamine-induced plasticity in major depressive disorder. Int J Neuropsychopharmacol.

[23] Eydipour Z, Nasehi M, Vaseghi S, Jamaldini SH, Zarrindast MR (2020). The role of 5-HT4 serotonin re-ceptors in the CA1 hippocampal region on memory acquisition impairment induced by total (TSD) and REM sleep deprivation (RSD). Physiol Behav.

[24] Florian C, Vecsey CG, Halassa MM, Haydon PG, Abel T (2011). Astrocyte-derived adenosine and A1 receptor activity contribute to sleep loss-induced deficits in hippocampal synaptic plasticity and memory in mice. J Neurosci.

[25] Fujihara H, Sei H, Morita Y, Ueta Y, Morita K (2003). Short-term sleep disturbance enhances brain-derived neurotrophic factor gene expression in rat hippocampus by acting as internal stressor. J Mol Neurosci.

[26] Gachon F, Nagoshi E, Brown SA, Ripperger J, Schibler U (2004). The mammalian circadian timing system: from gene expression to physiology. Chromosoma.

[27] Genzel L, Dresler M, Wehrle R, Grozinger M, Steiger A (2009). Slow wave sleep and REM sleep awakenings do not affect sleep dependent memory consolidation. Sleep.

[28] Genzer Y, Dadon M, Burg C, Chapnik N, Froy O (2016). Effect of dietary fat and the circadian clock on the expression of brain-derived neurotrophic factor (BDNF). Mol Cell Endocrinol.

[29] Giacobbo BL, Correa MS, Vedovelli K, de Souza CE, Spitza LM, Goncalves L (2016). Could BDNF be involved in compensatory mechanisms to maintain cognitive performance despite acute sleep deprivation? An exploratory study. Int J Psychophysiol.

[30] Gisquet-Verrier P, Smith C (1989). Avoidance performance in rat enhanced by postlearning paradoxical sleep deprivation. Behav Neural Biol.

[31] Grassi Zucconi G, Cipriani S, Balgkouranidou I, Scattoni R (2006). 'One night' sleep deprivation stimulates hippocampal neurogenesis. Brain Res Bull.

[32] Graves LA, Heller EA, Pack AI, Abel T (2003). Sleep deprivation selectively impairs memory consolidation for contextual fear conditioning. Learn Mem.

[33] Guo Z, Jiang Z, Jiang B, McClure MA, Mu Q (2019). High-frequency repetitive transcranial magnetic stimulation could improve impaired working memory induced by sleep deprivation. Neural Plast.

[34] Guzman-Marin R, Suntsova N, Methippara M, Greiffenstein R, Szymusiak R, McGinty D (2005). Sleep deprivation suppresses neurogenesis in the adult hippocampus of rats. Eur J Neurosci.

[35] Haack M, Sanchez E, Mullington JM (2007). Elevated inflammatory markers in response to prolonged sleep restriction are associated with increased pain experience in healthy volunteers. Sleep.

[36] Hairston IS, Little MT, Scanlon MD, Barakat MT, Palmer TD, Sapolsky RM (2005). Sleep restriction suppresses neurogenesis induced by hippocampus-dependent learning. J Neurophysiol.

[37] Hairston IS, Peyron C, Denning DP, Ruby NF, Flores J, Sapolsky RM (2004). Sleep deprivation effects on growth factor expression in neonatal rats: a potential role for BDNF in the mediation of delta power. J Neurophysiol.

[38] Havekes R, Abel T (2017). The tired hippocampus: the molecular impact of sleep deprivation on hippocampal function. Curr Opin Neurobiol.

[39] Havekes R, Bruinenberg VM, Tudor JC, Ferri SL, Baumann A, Meerlo P (2014). Transiently increasing cAMP levels selectively in hippocampal excitatory neurons during sleep deprivation prevents memory deficits caused by sleep loss. J Neurosci.

[40] Havekes R, Park AJ, Tudor JC, Luczak VG, Hansen RT, Ferri SL (2016). Sleep deprivation causes memory deficits by negatively impacting neuronal connectivity in hippocampal area CA1. Elife.

[41] Hennecke E, Lange D, Steenbergen F, Fronczek-Poncelet J, Elmenhorst D, Bauer A (2020). Adverse interaction effects of chronic and acute sleep deficits on spatial working memory but not on verbal working memory or declarative memory. J Sleep Res.

[42] Hussaini SA, Bogusch L, Landgraf T, Menzel R (2009). Sleep deprivation affects extinction but not acquisition memory in honeybees. Learn Mem.

[43] Hwang L, Ko IG, Jin JJ, Kim SH, Kim CJ, Chang B (2019). Dexmedetomidine ameliorates memory impairment in sleep-deprived mice. Anim Cells Syst (Seoul).

[44] Ishikawa A, Kanayama Y, Matsumura H, Tsuchimochi H, Ishida Y, Nakamura S (2006). Selective rapid eye movement sleep deprivation impairs the maintenance of long-term potentiation in the rat hippocampus. Eur J Neurosci.

[45] Javad-Moosavi BZ, Nasehi M, Vaseghi S, Jamaldini SH, Zarrindast MR (2020). Activation and inactivation of nicotinic receptnors in the dorsal hippocampal region restored negative effects of total (TSD) and REM Sleep Deprivation (RSD) on memory acquisition, locomotor activity and pain perception. Neuroscience.

[46] Javad-Moosavi BZ, Vaezi G, Nasehi M, Haeri-Rouhani SA, Zarrindast MR (2017). Critical role of CA1 muscarinic receptors on memory acquisition deficit induced by total (TSD) and REM sleep deprivation (RSD). Prog Neuropsychopharmacol Biol Psychiatry.

[47] Jiang Y, Zhu J (2015). Effects of sleep deprivation on behaviors and abnormal hippocampal BDNF/miR-10B expression in rats with chronic stress depression. Int J Clin Exp Pathol.

[48] Junek A, Rusak B, Semba K (2010). Short-term sleep deprivation may alter the dynamics of hippocampal cell proliferation in adult rats. Neuroscience.

[49] Kavcic P, Rojc B, Dolenc-Groselj L, Claustrat B, Fujs K, Poljak M (2011). The impact of sleep deprivation and nighttime light exposure on clock gene expression in humans. Croat Med J.

[50] Kim SE, Ko IG, Ji ES, Jin JJ, Hwang L, Kim SH (2019). Treadmill exercise alleviates circadian rhythm disruption-induced memory deficits by activation of glucocorticoid receptor and brain-derived neurotrophic factor-dependent pathway. Int Neurourol J.

[51] Kordestani-Moghadam P, Nasehi M, Khodagholi F, Vaseghi S, Zarrindast MR, Khani M (2020). The fluctuations of metabotropic glutamate receptor subtype 5 (mGluR5) in the amygdala in fear conditioning model of male Wistar rats following sleep deprivation, reverse circadian and napping. Brain Res.

[52] Kordestani-Moghadam P, Nasehi M, Vaseghi S, Khodagholi F, Zarrindast MR (2020). The role of sleep disturbances in depressive-like behavior with emphasis on alpha-ketoglutarate dehydrogenase activity in rats. Physiol Behav.

[53] Korte M, Carroll P, Wolf E, Brem G, Thoenen H, Bonhoeffer T (1995). Hippocampal long-term potentiation is impaired in mice lacking brain-derived neurotrophic factor. Proc Natl Acad Sci U S A.

[54] Korte M, Griesbeck O, Gravel C, Carroll P, Staiger V, Thoenen H (1996). Virus-mediated gene transfer into hippocampal CA1 region restores long-term potentiation in brain-derived neurotrophic factor mutant mice. Proc Natl Acad Sci U S A.

[55] Krause AJ, Simon EB, Mander BA, Greer SM, Saletin JM, Goldstein-Piekarski AN (2017). The sleep-deprived human brain. Nat Rev Neurosci.

[56] Kreutzmann JC, Havekes R, Abel T, Meerlo P (2015). Sleep deprivation and hippocampal vulnerability: changes in neuronal plasticity, neurogenesis and cognitive function. Neuroscience.

[57] Krueger JM (2008). The role of cytokines in sleep regulation. Curr Pharm Des.

[58] Kuhn M, Wolf E, Maier JG, Mainberger F, Feige B, Schmid H (2016). Sleep recalibrates homeostatic and associative synaptic plasticity in the human cortex. Nat Commun.

[59] Liang FQ, Allen G, Earnest D (2000). Role of brain-derived neurotrophic factor in the circadian regulation of the suprachiasmatic pacemaker by light. J Neurosci.

[60] Liang FQ, Sohrabji F, Miranda R, Earnest B, Earnest D (1998). Expression of brain-derived neurotrophic factor and its cognate receptor, TrkB, in the rat suprachiasmatic nucleus. Exp Neurol.

[61] Liang FQ, Walline R, Earnest DJ (1998). Circadian rhythm of brain-derived neurotrophic factor in the rat suprachiasmatic nucleus. Neurosci Lett.

[62] Lopez-Virgen V, Zarate-Lopez D, Adirsch FL, Collas-Aguilar J, Gonzalez-Perez O (2015). Gac Med Mex.

[63] Ma X, Liu L, Meng J (2017). MicroRNA-125b promotes neurons cell apoptosis and Tau phosphorylation in Alzheimer's disease. Neurosci Lett.

[64] Mahboubi S, Nasehi M, Imani A, Sadat-Shirazi MS, Zarrindast MR, Vousooghi N (2019). Benefit effect of REM-sleep deprivation on memory impairment induced by intensive exercise in male wistar rats: with respect to hippocampal BDNF and TrkB. Nat Sci Sleep.

[65] Mahdavi MS, Nasehi M, Vaseghi S, Mousavi Z, Zarrindast MR (2020). The effect of alpha lipoic acid on passive avoidance and social interaction memory, pain perception, and locomotor activity in REM sleep-deprived rats. Pharmacol Rep.

[66] Mascetti L, Foret A, Schrouff J, Muto V, Dideberg V, Balteau E (2013). Concurrent synaptic and systems memory consolidation during sleep. J Neurosci.

[67] McDermott CM, LaHoste GJ, Chen C, Musto A, Bazan NG, Magee JC (2003). Sleep deprivation causes behavioral, synaptic, and membrane excitability alterations in hippocampal neurons. J Neurosci.

[68] Medic G, Wille M, Hemels ME (2017). Short- and long-term health consequences of sleep disruption. Nat Sci Sleep.

[69] Meerlo P, Mistlberger RE, Jacobs BL, Heller HC, McGinty D (2009). New neurons in the adult brain: the role of sleep and consequences of sleep loss. Sleep Med Rev.

[70] Moldovan M, Constantinescu AO, Balseanu A, Oprescu N, Zagrean L, Popa-Wagner A (2010). Sleep deprivation attenuates experimental stroke severity in rats. Exp Neurol.

[71] Montes-Rodriguez CJ, Rueda-Orozco PE, Prospero-Garcia O (2019). Total sleep deprivation impairs fear memory retrieval by decreasing the basolateral amygdala activity. Brain Res.

[72] Moravcova S, Cervena K, Mikova H, Pacesova D, Pallag G, Novotny J (2020). Social defeat stress affects resident's clock gene and bdnf expression in the brain. Stress.

[73] Morgenthaler J, Wiesner CD, Hinze K, Abels LC, Prehn-Kristensen A, Goder R (2014). Selective REM-sleep deprivation does not diminish emotional memory consolidation in young healthy subjects. PLoS One.

[74] Motomura Y, Kitamura S, Oba K, Terasawa Y, Enomoto M, Katayose Y (2013). Sleep debt elicits negative emotional reaction through diminished amygdala-anterior cingulate functional connectivity. PLoS One.

[75] Motomura Y, Kitamura S, Oba K, Terasawa Y, Enomoto M, Katayose Y (2014). Sleepiness induced by sleep-debt enhanced amygdala activity for subliminal signals of fear. BMC Neurosci.

[76] Mueller AD, Meerlo P, McGinty D, Mistlberger RE (2015). Sleep and adult neurogenesis: implications for cognition and mood. Curr Top Behav Neurosci.

[77] Nasehi M, Shirkhodaei A, Ebrahimi-Ghiri M, Zarrindast MR (2019). Abolishment of fear memory-disruptive effects REM sleep deprivation by harmane. Biomed Pharmacother.

[78] Nilsonne G, Lekander M, Akerstedt T, Axelsson J, Ingre M (2016). Diurnal variation of circulating interleukin-6 in humans: a meta-analysis. PLoS One.

[79] Ocalan B, Cakir A, Koc C, Suyen GG, Kahveci N (2019). Uridine treatment prevents REM sleep deprivation-induced learning and memory impairment. Neurosci Res.

[80] Olonode ET, Aderibigbe AO, Adeoluwa OA, Eduviere AT, Ben-Azu B (2019). Morin hydrate mitigates rapid eye movement sleep deprivation-induced neurobehavioural impairments and loss of viable neurons in the hippocampus of mice. Behav Brain Res.

[81] Patterson SL, Abel T, Deuel TA, Martin KC, Rose JC, Kandel ER (1996). Recombinant BDNF rescues deficits in basal synaptic transmission and hippocampal LTP in BDNF knockout mice. Neuron.

[82] Payne JL, Quiroz JA, Zarate CA, Manji HK (2002). Timing is everything: does the robust upregulation of noradrenergically regulated plasticity genes underlie the rapid antidepressant effects of sleep deprivation?. Biol Psychiatry.

[83] Peng Y, Wang W, Tan T, He W, Dong Z, Wang YT (2016). Maternal sleep deprivation at different stages of pregnancy impairs the emotional and cognitive functions, and suppresses hippocampal long-term potentiation in the offspring rats. Mol Brain.

[84] Peng Z, Dai C, Ba Y, Zhang L, Shao Y, Tian J (2020). Effect of sleep deprivation on the working memory-related N2-P3 components of the event-related potential waveform. Front Neurosci.

[85] Prince TM, Wimmer M, Choi J, Havekes R, Aton S, Abel T (2014). Sleep deprivation during a specific 3-hour time window post-training impairs hippocampal synaptic plasticity and memory. Neurobiol Learn Mem.

[86] Rahmani M, Rahmani F, Rezaei N (2020). The brain-derived neurotrophic factor: missing link between sleep deprivation, insomnia, and depression. Neurochem Res.

[87] Rajizadeh MA, Esmaeilpour K, Haghparast E, Ebrahimi MN, Sheibani V (2020). Voluntary exercise modulates learning & memory and synaptic plasticity impairments in sleep deprived female rats. Brain Res.

[88] Randazzo AC, Muehlbach MJ, Schweitzer PK, Walsh JK (1998). Cognitive function following acute sleep restriction in children ages 10-14. Sleep.

[89] Rangtell FH, Karamchedu S, Andersson P, Liethof L, Olaya Bucaro M, Lampola L (2019). A single night of sleep loss impairs objective but not subjective working memory performance in a sex-dependent manner. J Sleep Res.

[90] Redwine L, Hauger RL, Gillin JC, Irwin M (2000). Effects of sleep and sleep deprivation on interleukin-6, growth hormone, cortisol, and melatonin levels in humans. J Clin Endocrinol Metab.

[91] Rezaie M, Nasehi M, Vaseghi S, Alimohammadzadeh K, Islami Vaghar M, Mohammadi-Mahdiabadi-Hasani MH (2020). The interaction effect of sleep deprivation and cannabinoid type 1 receptor in the CA1 hippocampal region on passive avoidance memory, depressive-like behavior and locomotor activity in rats. Behav Brain Res.

[92] Rezaie M, Nasehi M, Vaseghi S, Mohammadi-Mahdiabadi-Hasani MH, Zarrindast MR, Nasiri Khalili MA (2020). The protective effect of alpha lipoic acid (ALA) on social interaction memory, but not passive avoidance in sleep-deprived rats. Naunyn Schmiedebergs Arch Pharmacol.

[93] Rosales-Lagarde A, Armony JL, Del Rio-Portilla Y, Trejo-Martinez D, Conde R, Corsi-Cabrera M (2012). Enhanced emotional reactivity after selective REM sleep deprivation in humans: an fMRI study. Front Behav Neurosci.

[94] Salehpour F, Farajdokht F, Erfani M, Sadigh-Eteghad S, Shotorbani SS, Hamblin MR (2018). Transcranial near-infrared photobiomodulation attenuates memory impairment and hippocampal oxidative stress in sleep-deprived mice. Brain Res.

[95] Sauvet F, Arnal PJ, Tardo-Dino PE, Drogou C, Van Beers P, Erblang M (2020). Beneficial effects of exercise training on cognitive performances during total sleep deprivation in healthy subjects. Sleep Med.

[96] Sharma R, Sahota P, Thakkar MM (2020). Short-term sleep deprivation immediately after contextual conditioning inhibits BDNF signaling and disrupts memory consolidation in predator odor trauma mice model of PTSD. Brain Res.

[97] Shearman LP, Sriram S, Weaver DR, Maywood ES, Chaves I, Zheng B (2000). Interacting molecular loops in the mammalian circadian clock. Science.

[98] Shors TJ (2001). Acute stress rapidly and persistently enhances memory formation in the male rat. Neurobiol Learn Mem.

[99] Silva RH, Abilio VC, Takatsu AL, Kameda SR, Grassl C, Chehin AB (2004). Role of hippocampal oxidative stress in memory deficits induced by sleep deprivation in mice. Neuropharmacology.

[100] Silvestri AJ (2005). REM sleep deprivation affects extinction of cued but not contextual fear conditioning. Physiol Behav.

[101] Silvestri AJ, Root DH (2008). Effects of REM deprivation and an NMDA agonist on the extinction of conditioned fear. Physiol Behav.

[102] Smith C, Rose GM (1996). Evidence for a paradoxical sleep window for place learning in the Morris water maze. Physiol Behav.

[103] Tabassum S, Misrani A, Tang BL, Chen J, Yang L, Long C (2019). Jujuboside A prevents sleep loss-induced disturbance of hippocampal neuronal excitability and memory impairment in young APP/PS1 mice. Sci Rep.

[104] Taishi P, Sanchez C, Wang Y, Fang J, Harding JW, Krueger JM (2001). Conditions that affect sleep alter the expression of molecules associated with synaptic plasticity. Am J Physiol Regul Integr Comp Physiol.

[105] Tang H, Li K, Dou X, Zhao Y, Huang C, Shu F (2020). The neuroprotective effect of osthole against chronic sleep deprivation (CSD)-induced memory impairment in rats. Life Sci.

[106] Tantawy AO, Tallawy HN, Farghaly HR, Farghaly WM, Hussein AS (2013). Impact of nocturnal sleep deprivation on declarative memory retrieval in students at an orphanage: a psychoneuroradiological study. Neuropsychiatr Dis Treat.

[107] Tian S, Huang F, Li P, Ouyang X, Li Z, Deng H (2009). Rapid eye movement sleep deprivation does not affect fear memory reconsolidation in rats. Neurosci Lett.

[108] Tobler I, Borbely AA (1990). The effect of 3-h and 6-h sleep deprivation on sleep and EEG spectra of the rat. Behav Brain Res.

[109] Tudor JC, Davis EJ, Peixoto L, Wimmer ME, van Tilborg E, Park AJ (2016). Sleep deprivation impairs memory by attenuating mTORC1-dependent protein synthesis. Sci Signal.

[110] Vargas I, Payne JD, Muench A, Kuhlman KR, Lopez-Duran NL (2019). Acute sleep deprivation and the selective consolidation of emotional memories. Learn Mem.

[111] Vecsey CG, Baillie GS, Jaganath D, Havekes R, Daniels A, Wimmer M (2009). Sleep deprivation impairs cAMP signalling in the hippocampus. Nature.

[112] Vecsey CG, Peixoto L, Choi JH, Wimmer M, Jaganath D, Hernandez PJ (2012). Genomic analysis of sleep deprivation reveals translational regulation in the hippocampus. Physiol Genomics.

[113] Vgontzas AN, Zoumakis M, Papanicolaou DA, Bixler EO, Prolo P, Lin HM (2002). Chronic insomnia is associated with a shift of interleukin-6 and tumor necrosis factor secretion from nighttime to day-time. Metabolism.

[114] Voderholzer U, Piosczyk H, Holz J, Landmann N, Feige B, Loessl B (2011). Sleep restriction over several days does not affect long-term recall of declarative and procedural memories in adolescents. Sleep Med.

[115] Wadhwa M, Prabhakar A, Anand JP, Ray K, Prasad D, Kumar B (2019). Complement activation sustains neuroinflammation and deteriorates adult neurogenesis and spatial memory impairment in rat hippocampus following sleep deprivation. Brain Behav Immun.

[116] Wadhwa M, Prabhakar A, Ray K, Roy K, Kumari P, Jha PK (2017). Inhibiting the microglia activation improves the spatial memory and adult neurogenesis in rat hippocampus during 48 h of sleep deprivation. J Neuroinflammation.

[117] Wang GP, Huang LQ, Wu HJ, Zhang L, You ZD, Zhao ZX (2009). Calcineurin contributes to spatial memory impairment induced by rapid eye movement sleep deprivation. Neuroreport.

[118] Wang H, Liu Y, Briesemann M, Yan J (2010). Computational analysis of gene regulation in animal sleep deprivation. Physiol Genomics.

[119] Wang S, Su G, Zhang Q, Zhao T, Liu Y, Zheng L (2018). Walnut (Juglans regia) peptides reverse sleep deprivation-induced memory impairment in rat via alleviating oxidative stress. J Agric Food Chem.

[120] Wang W, Yang L, Liu T, Wang J, Wen A, Ding Y (2020). Ellagic acid protects mice against sleep deprivation-induced memory impairment and anxiety by inhibiting TLR4 and activating Nrf2. Aging (Albany NY).

[121] Weil ZM, Norman GJ, Karelina K, Morris JS, Barker JM, Su AJ (2009). Sleep deprivation attenuates inflammatory responses and ischemic cell death. Exp Neurol.

[122] Yang SQ, Jiang L, Lan F, Wei HJ, Xie M, Zou W (2019). Inhibited endogenous H2S generation and excessive autophagy in hippocampus contribute to sleep deprivation-induced cognitive impairment. Front Psychol.

[123] Yoo SS, Gujar N, Hu P, Jolesz FA, Walker MP (2007). The human emotional brain without sleep - a prefrontal amygdala disconnect. Curr Biol.

[124] Zagaar M, Dao A, Levine A, Alhaider I, Alkadhi K (2013). Regular exercise prevents sleep deprivation associated impairment of long-term memory and synaptic plasticity in the CA1 area of the hippocampus. Sleep.

[125] Zhang J, Zhang L, Chang Y, Gu Q, Zhang J, Zhu Z (2020). The endocannabinoid system contributes to memory deficits induced by rapid-eye-movement sleep deprivation in adolescent mice. Neuroscience.

[126] Zhang L, Zhang HQ, Liang XY, Zhang HF, Zhang T, Liu FE (2013). Melatonin ameliorates cognitive impairment induced by sleep deprivation in rats: role of oxidative stress, BDNF and CaMKII. Behav Brain Res.

[127] Zhang YW, Li XQ, Tan WF, Fang B, Ma H (2020). Postoperative 24-h acute sleep deprivation improves learning and memory through inhibition of tau phosphorylation in the hippocampal neurons of splenectomized rats. Nat Sci Sleep.

[128] Zhao Z, Huang L, Wu H, Li Y, Zhang L, Yin Y (2010). Neuropeptide S mitigates spatial memory impairment induced by rapid eye movement sleep deprivation in rats. Neuroreport.

[129] Zhou HR, Wu JR, Bei L, Wang BX, Xu H, Wang JT (2020). Hydroalcoholic extract from Abelmoschus manihot (Linn.) Medicus flower reverses sleep deprivation-evoked learning and memory deficit. Food Funct.

